# Italian validation of the health education impact questionnaire (heiQ) in people with chronic conditions

**DOI:** 10.1186/s12955-020-01329-9

**Published:** 2020-03-30

**Authors:** Andrea Pozza, Richard H. Osborne, Gerald R. Elsworth, Fabio Ferretti, Anna Coluccia

**Affiliations:** 1 Department of Medical Sciences, Surgery and Neurosciences, University of Siena, Viale Bracci, 16 53100 Siena, Italy; 2 Centre for Global Health and Equity, Swinburne University of Technology, Melbourne, Australia; 3 University of Copenhagen, Copenhagen, Denmark

**Keywords:** Chronic diseases, Self-management, Health education, Psychometric properties, Perceived health status, Quality of life, Diabetes, Cardiovascular diseases

## Abstract

**Background:**

The health education impact Questionnaire (heiQ) measures eight self-management skills in people with chronic conditions. It seems to be important to provide cross-cultural evidence on its properties in non-English healthcare contexts. The present study assessed the psychometric properties of the heiQ in Italian adults with chronic conditions.

**Methods:**

Two hundred ninety-nine individuals with a chronic condition (mean age = 61.4 years, 50.16% females) completed the heiQ and the Medical Outcomes Study-Short Form (SF-36). Confirmatory factor analyses, Composite Reliability Indices, and bivariate correlations were performed.

**Results:**

Structural validity based on 8 correlated factors with good fit was in line with previous research. Concurrent validity was confirmed, as shown by moderate associations between the scores on the Constructive attitudes and approaches, Self-monitoring and insight, Health directed activities, Social integration and support, and Emotional distress subscales and the scores on SF-36 Physical functioning, General health perceptions, Vitality, Social functioning, Perceived mental health and Role limitations due to physical and emotional problems subscales.

**Conclusions:**

The Italian heiQ has strong properties and it can be used routinely also in the Italian healthcare services.

## Background

The increasing number of individuals with chronic diseases is a challenge for health care systems that requires a radical change in the health paradigm including a more active involvement of patients in the care process, the self-management of their disease, and greater attention to their perception of health status [1–5]. Consequently, reliable tools are essential to allow practitioners to assess key variables implicated in the improvement of these outcomes [6–12].

In Australia, Osborne and colleagues developed the health education impact Questionnaire (heiQ) [6] a self-report questionnaire, which assesses eight independent key self-management skills for adults with chronic conditions. These skills include Positive and active engagement in life (i.e., motivation to be active and engage in life-fulfilling activities), Health-directed activities (healthful behaviours including prevention, diet, and exercise), Skill and technique acquisition (knowledge-based skills and techniques including the use of aids that help individuals manage symptoms and health problems), Constructive attitudes and approaches (attempts to minimize the effects of illness and determination not to allow the illness control their life), Self-monitoring and insight (the individuals’ ability to monitor their condition, and their physical and/or emotional responses that leads to insight and appropriate action/s to self-manage), Health service navigation (an individual’s understanding of and ability to interact with a range of health organizations and health professionals), Social integration and support (the positive impact of social engagement and support that evolves through interaction with others and the impact may arise from interaction with others sharing similar health-related life experiences), and Emotional distress (overall negative affective responses to illness, including anxiety, anger, and depression attributed to the illness).

The measure was originally developed to detect the benefits of health education programs for a wide range of patient groups and specifically sought to provide useful information to medical staff, researchers and policymakers. The items, which were developed from the patients’ perspective and evaluated with a Likert response format, have been found to have strong properties using a range of psychometric methods [9, 10]. Since its development, the heiQ has undergone minor refinements. The original version [6] had 42 items with a 6-point response format. Analyses during the construction of the questionnaire suggested that some items had disordered thresholds: some respondents were unable to differentiate between the two midpoints “slightly agree” and “slightly disagree”. Further analyses also suggested that two items could be removed without compromising content validity. Thus, the response format was simplified to a 4-point scale (“strongly disagree” to “strongly agree”) and 2 items were removed [6].

Since concepts related to health can be hypothesized to depend on socio-cultural aspects, the heiQ has been translated and adopted in different healthcare and socio-cultural contexts, including Canada, Germany, France, the Netherlands, Norway, and Japan [7–12].

### Rationale

It is important to provide further evidence on the properties of the heiQ in non-English healthcare contexts, as the questionnaire is being used in international cross-cultural research [13]. Despite the growing evidence on its structural validity and reliability in different socio-cultural contexts, there is a lack of tools measuring self-management skills in Italy. Given the lack of attention Italian practitioners have paid to self-management skills to date, patients in Italy are failing to keep up with international evidence and the move to more patient-centred healthcare [14, 15]. Moreover, concurrent validity measured by the relation between the heiQ self-management skills and perceived health status has been explored by only a study [11] which used the Medical Outcomes Survey Short Form-36 (SF-36) [16].

### Objectives and hypotheses

The present study aimed to provide further evidence on the psychometric properties of the heiQ among Italian adults with chronic conditions by assessing structural validity, reliability, convergent/divergent and concurrent validity.

Convergent and divergent validity was investigated by exploring the associations between the self-management skills. In line with previous data [11], it was hypothesized to obtain a) moderate correlations between Positive and active engagement in life, Constructive attitudes and approaches, and Social integration and support, b) moderate associations between Skill and technique acquisition and Health service navigation and c) weak associations between Emotional distress and all the other self-management skills.

Finally, concurrent validity was verified by investigating the associations between self-management skills and outcomes of perceived health status, as measured by the SF-36 subscales (i.e., Physical Functioning, Role Limitations due to Physical Problems, Bodily Pains, General Health Perceptions, Vitality, Social Functioning, Role Limitations due to Emotional Problems, and Perceived Mental Health). Perceived health status was defined as the perception of a state of complete physical, mental and social well-being, and not merely the absence of disease and infirmity [17]. Based on previous data [11], it was expected that a) physical functioning correlates moderately with emotional distress, b) role limitations due to physical problems correlates moderately with emotional distress, c) bodily pain correlates moderately with emotional distress, d) general health perceptions, vitality, social functioning and perceived mental health correlate moderately with positive and active engagement in life, constructive attitudes and approaches, social integration and support and emotional distress, e) role limitations due to emotional problems correlate moderately with positive and active engagement in life, constructive attitudes and approaches, and emotional distress.

## Methods

The study included two phases. First, the original heiQ was translated and culturally adapted in Italian, following the standardised protocol of the Deakin University [18]. In the second phase, the psychometric properties of the measure were investigated in a large group of adults with chronic diseases.

### Translation and cultural adaptation of the heiQ

The translation and cultural adaptation process consisted of a first step with a forward translation by a professional, licensed translator as requested in the protocol (Italian-English bilingual, native Italian speaker) who followed an item intent guide, a second phase with a critical review by the Italian research staff (AP, FF, AC), and finally a blinded independent back translation (Italian-English bilingual, native English speaker). Apart from the translation, items were reviewed for cultural appropriateness. The meaning of every statement in the final translation was checked with one of the original authors (RHO) against all previous language versions through written reports and a consensus online meeting.

### Participants

Two hundred ninety-nine individuals with a diagnosis of a chronic condition participated (Table 1). Mean age was 61.4 years old (SD = 13.4, range = 23–86), about 50% of the sample were females. Diagnosis duration was 9.57 years (SD = 7.49).

**Table 1:** Socio-demographics and clinical features (*n* = 299)

	n	%	M (SD; range)
Female gender	150	50.16	
Age (years)			61.4 (13.4; 23–86)
**Sites**			
Santa Maria alle Scotte University Hospital of Siena	181	60.53	
Azienda USL Toscana Sud Est	61	20.40	
Casa Sollievo della Soffferenza Hospital of S. Giovanni Rotondo	32	10.70	
Azienda USL Toscana Centro	25	8.36	
**Chronic diseases**			
Arthritis	50	16.72	
Obstructive Pulmonary Disease Chronic Bronchitis	6	2	
Diabetes	133	44.48	
Cardiovascular diseases	14	4.68	
Kidney diseases	18	6.02	
Hypertension	32	10.70	
Systemic Schlerosis	19	6.35	
Other diseases	27	9.03	
Time since the diagnosis (years)			9.57 (7.49; 0.8–40)
Presence of polypathologies of chronic diseases	16	5.35	

Participants were recruited in outpatient wards at a University Hospital and at community healthcare outpatient services. Data were collected from October 2013 to May 2014. All the participants completed the questionnaires individually with the assistance of a psychologist, who provided information about the aims of the study. In accordance with the Ethical Principles of Psychologists and Code of Conduct [19], all the participants, who were included, provided written informed consent to participate in the study after having received a detailed description of the aims.

### The health education impact questionnaire (heiQ)

The heiQ is a self-report measure which covers eight dimensions, each one representing specific self-management capacities and skills: 1. Positive and active engagement in life, 2. Health directed activities, 3. Skill and technique acquisition, 4. Constructive attitudes and approaches, 5. Self-monitoring and insight, 6. Health service navigation, 7. Social integration and support, and 8. Emotional distress. Each dimension is assessed by between four and six questions, with forty questions overall. Responses are marked on a 4-point Likert scale, using the endpoints “strongly disagree/strongly agree”. The scale scores are formed by computing the mean of respective items. A higher score indicates stronger skills in the management of the chronic condition, except in the emotional distress dimension, which is negatively scored. Missing values were replaced with the mean values obtained by the individuals.

### Medical outcomes survey short Form-36 (SF-36)

The SF-36 [16] was used as a measure of perceived health status to assess concurrent validity of the heiQ scales. The SF-36 is a generic measure of perceived health status which assesses eight dimensions: Physical Functioning (10 items), Role Limitations due to Physical Problems (4 items), Bodily Pains (2 items), General Health Perceptions (5 items), Vitality (4 items), Social Functioning (2 items), Role Limitations due to Emotional Problems (2 items), and Perceived Mental Health (5 items). In addition, a single item provides an indication of perceived change in general health status over a one-year time period.

According to previous literature reviews on this concept, the SF-36 can be considered a gold standard measure of perceived health status, which can be used to assess concurrent validity since it describes health using functioning and well-being [20, 21]. The scales of the Italian version [22] have demonstrated good internal consistency with Cronbach’s alpha ranging from .78 to .93. In the present study, the Cronbach alpha of the scales ranged between .81 and .91.

### Data analysis

The analysis was conducted using the SPSS software version 21.00 and the AMOS software. Descriptive statistics were generated for each item to explore floor and ceiling effects and to determine the extent of missing values. Distributional properties of the heiQ items were evaluated through skewness and kurtosis indices. Subsequently, to assess structural validity, confirmatory factor analysis (CFA) was performed through structural equation modelling [23]. Following the factor structure reported in the original validation study [6], an a-priori model was tested with eight correlated factors. To evaluate goodness of fit of the model, the following indices recommended by Hu and Bentler [24] were used: the Comparative Fit Index (CFI), the Tucker Lewis Index (TLI): values ranging from .95 and 1 indicate excellent fit, from .90 and .95 good fit. In addition, the Root Mean Square Residual (RMR) was considered: values less than 0.08 indicate acceptable fit, and those less than .05 good fit. The Root Mean Square Error of Approximation (RMSEA) was also used as fit index: values less than .06 suggest good fit.

Reliability was examined as internal consistency through the calculation of composite reliability indices (CRI), in order to compare the results of the Italian heiQ with those reported in previous validation studies on the measure. CRI values can be interpreted like Cronbach alpha. In the current study, CRI values were assessed according to Nunnally and Bernstein’s guidelines [25] (CRI > .70 = acceptable, CRI > .80 = good, CRI > .90 = excellent).

Convergent and divergent validity was examined computing Pearson’s bivariate correlations between the heiQ subscale scores. Finally, concurrent validity was tested by calculating Pearson’s bivariate correlations between the heiQ subscale scores and the SF-36 subscale scores. Values on the correlation coefficients were interpreted according to the following criteria [26]: 0 < | r | < .30 = weak; .30 < | r | < .50 = moderate; .50 < | r | < .70 = strong; .70 < | r | < 1 = very strong. Power calculations were run for the correlational analysis: for a medium effect size, 80% power, and a significance set at *p* < .05, the required sample size for bivariate correlations was at least 162 participants.

## Results

### Structural validity

Missing values (3–7%) were handled by replacing the missing values with the mean values reported by the individuals. The assumption of distribution normality was satisfied since the ratio between kurtosis or skewness and their standard errors fell within the requested range between − 1 and + 1. Floor and ceiling effects were not found since the proportions of participants reporting the lowest or the highest scores were lower than 15%, as requested by the COSMIN guidelines.

First, a model with eight correlated factors was tested. However, despite values on the χ^2^/df was in the range between 1 and 3 and values on the RMSEA and the RMR were lower than .06, suggesting good fit, values on the CFI and TLI were still not acceptable, evidencing partially inadequate fit. Subsequently, to further improve the model fit, modification indices were inspected and covariances between residuals of some items were added. In particular, covariances were added between the residuals of the item 15 (“I feel like I am actively involved in life”) loading on 1. Positive and active engagement in life and those of the item 8 (“I am doing interesting things in my life”), loading on the same factor. Covariances were added between the residuals of the item 3 (“As well as seeing my doctor, I regularly monitor changes”) and those of the item 17 (“I carefully watch my health and do what is necessary to keep”), which both loaded on 5. Self-monitoring and insight. Covariances were added also between the residuals of the item 3 and those of the item 37 (“I get enough chances to talk about my health”). Finally, covariances were added between the residuals of the item 34 (“My health problems do not ruin my life”) and those of the item 39 (“I do not let my health problems control my life”), which both loaded on 4. Constructive attitudes and approaches. Covariances were added between the residuals of the item 32 (“I confidently give healthcare professionals the information”) loading on 6. Health service navigation and those of the item 16 (“When I have health problems, I have a clear understanding of what I need to do”) loading on 5. Self-monitoring and insight. This model with the inclusion of covariances between residuals of some items showed improved fit in all the indices as compared with the previous models. Fit indices and the comparisons with previous validation studies are presented in Table 2.

**Table 2:** Model fit indices of the heiQ in the present study (*n* = 299) and in previous studies

Models tested in the present study	χ^**2**^	χ^**2**^/df	CFI	TLI	RMSEA	RMR
Correlated factors	1183.74*	1.75	.88	.87	.05	.05
Correlated factors with covariances between residuals	1094.28*	1.63	.91	.90	.04	.05
**Fit indices in previous studies**						
Original Australian study [7]	1420*	1.80	.99	n.r.	.04	n.r
Dutch study [8]	1257.77*	1.76	.89	.88	.05	n.r
French study [9]	1210.15*	1.69	.98	.91	.06	n.r
German study [11]	2223.96*	3.32	.92	n.r.	.04	n.r

*Note.* * *p* < .001. n.r. = not reported in the study paper. *CFI* Comparative Fit Index; *heiQ* health education impact Questionnaire; *GFI* Goodness of Fit Index; *RMR* Root Mean Residual; *RMSEA* Root Mean Squared Error Residual Approximation; Tucker-Lewis Index

### Reliability

Reliability was investigated by the calculation of CRI values. Findings showed that CRI were acceptable to good for all the subscales according to the criteria proposed by Nunnally and Bernstein [25]. On one hand, values were comparable with those reported in the Australian study [6] for 2. Health directed activities, 3. Skill and technique acquisition, 8. Emotional distress. On the other hand, values were lower (acceptable instead of good) for 1. Positive and active engagement in life, 4. Constructive attitudes and approaches, 6. Health service navigation than the Australian study [6]. CRI values for all the heiQ subscales and the comparison with previous validation studies are provided in Table 3.

**Table 3:** Composite reliability indices of the heiQ in the present study (*n* = 299) and in previous studies

	Present study	Original Australian study [7]	Norwegian study [12]	French study [9]	Dutch study [8]
**heiQ subscales**	**CRI**	**CRI**	**CRI**	**CRI**	**CRI**
1.Positive and active engagement in life	.72	.83	.79	.86	.81
2.Health directed activities	.84	.82	.80	.87	.82
3.Skill and technique acquisition	.71	.73	.76	.81	.82
4.Constructive attitudes and approaches	.76	.83	.83	.81	.86
5.Self-monitoring and insight	.65	.70	.61	.69	.67
6.Health service navigation	.78	.82	.72	.80	.85
7.Social integration and support	.77	.88	.83	.87	.85
8.Emotional distress	.86	.88	.90	.87	.89

*Note*. *CRI* Composite Reliability Indices, *heiQ* health education impact Questionnaire

### Convergent and divergent validity

Bivariate correlations between scores on the heiQ subscales are presented in Table 4. Intercorrelations were all moderate between scores on five subscales (1. Positive and active engagement in life, 4. Constructive attitudes and approaches, 5. Self-monitoring and insight, 6. Skill and technique acquisition, 7. Social integration and support). Intercorrelations between scores on 2. Health directed activities subscale and the other subscales were moderate only with those on 1. Positive and active engagement in life subscale. Intercorrelations between scores on 8. Emotional distress subscale were moderate and negative only with those on 4. Constructive attitudes and approaches subscale.

**Table 4:** Convergent and divergent validity: correlations between the heiQ subcale scores (*n* = 299)

heiQ subcales	2.	3.	4.	5.	6.	7.	8.
1.Positive and active engagement in life	.35^*^	.55^*^	.51^**^	.45^*^	.43^*^	.41^*^	−.20^*^
2.Health directed activities		.27^*^	.19^*^	.28^*^	.11^*^	.21^*^	−.05
3.Skill and technique acquisition			.54^*^	.60^*^	.60^*^	.50^*^	−.13^*^
4.Constructive attitudes and approaches				.38^*^	.47^*^	.45^*^	−.35^*^
5.Self-monitoring and insight					.59^*^	.41^*^	.08
6.Health service navigation						.49^*^	−.04
7.Social integration and support							−.18^*^
8.Emotional distress							1

*Note.* *p < .01, ^**^p < .001; *heiQ* health education impact Questionnaire

### Concurrent validity

Bivariate correlations between scores on the heiQ and those on the SF-36 subscales are presented in Table 5. Moderate positive correlations were found between scores on 2. Health directed activities subscale and those on the SF-36 Vitality and Perceived mental health. Scores on 4. Constructive attitudes and approaches subscale moderately and positively correlated with those on all the SF-36 subscales, except for SF-36 Bodily pain. Scores on 5. Self-monitoring and insight subscale moderately and positively correlated with those on the SF-36 Physical functioning, SF-36 Vitality, and SF-36 Perceived mental health. Scores on 7. Social integration and support subscale moderately and positively correlated with those on the SF-36 Vitality and Perceived mental health. No correlation was found between scores on 7. Social integration and support subscale and SF-36 Social functioning. Finally, a weak correlation was found between scores on 8. Emotional distress and SF-36 Perceived mental health.

**Table 5:** Concurrent validity: correlations between the heiQ subcale scores and those on the SF-36 subscales (*n* = 299)

heiQ scales	SF-36 Physical functioning	SF-36 Role limitations due to physical problems	SF-36 Bodily pains	SF-36 General health perceptions	SF-36 Vitality	SF-36 Social functioning	SF-36 Role limitations due to emotional problems	SF-36 Perceived mental health
1. Positive and active engagement in life	.24^*^	.18	.10	.30^*^	.28^*^	.23^*^	.11	.30^*^
2. Health directed activities	.26^*^	.18	−.01	.16	.32^**^	.16	.20	.37^**^
3. Skill and technique acquisition	.23	.20	.09	.21	.20	.06	.14	.17
4. Constructive attitudes and approaches	.45^**^	.38^**^	.24^*^	.39^**^	.42^**^	.41^**^	.31^**^	.43^**^
5. Self-monitoring and insight	.33^**^	.21	.24^*^	.20	.35^**^	.12	.10	.36^**^
6. Health service navigation	.14	.20	.22	.14	.30^*^	.17	.07	.23
7. Social integration and support	.25^*^	.10	.12	.27^*^	.34^**^	.11	.12	.36^**^
8. Emotional distress	−.40^**^	−.23	−.27^*^	−.42^**^	−.25^*^	−.40^**^	−.25^*^	−.32^**^

*Note.* **p < .001; *p < .05; *heiQ* health education impact Questionnaire; *SF-36* Medical Outcomes Survey Short Form-36

## Discussion

### Summary of results

The present study investigated the psychometric properties of the Italian heiQ in adults with chronic conditions. Key findings included the evidence that a model with eight correlated dimensions showed good fit. A strength of this finding is that it further confirms that the heiQ has strong properties and can be used routinely also in the Italian healthcare contexts. In addition, evidence of good fit of the questionnaire is important in Italy, like in other European countries, where the relationship between the medical staff and the patients is still asymmetrical, and there is a need of tools with well-established properties capable to assess self-management skills. The inclusion of covariances between the residuals of some items improved the fit of the eight-correlated factor model. Covariances between within-scale residuals were added between the item 15 (“I feel like I am actively involved in life”) and the item 8 (“I am doing interesting things in my life”), both loading on 1. Positive and active engagement in life dimension. This improved result could be explained by the fact that the content of the two items is largely overlapping, since in the Italian language the translation of the statement “To be actively involved” is very similar to the translation of that one “To do interesting things”. Covariances were added also between the residuals of the item 32 (“I confidently give healthcare professionals the information”) loading on 6. Health service navigation and those of the item 16 (“When I have health problems, I have a clear understanding”) loading on 5. Self-monitoring and insight. It could be that for Italian patients, giving healthcare professionals information implies also having a clear understanding of their disease and symptoms, and maybe this overlapping between these two concepts could explain such result in the Italian version of the heiQ.

In addition, covariances were added also between the residuals of the item 3 (“As well as seeing my doctor, I regularly monitor changes in my health”) and those of the item 17 (“I carefully watch my health and do what is necessary to keep healthy”), which both loaded on the 5. Self-monitoring and insight. Probably, this overlap could be attributed to the presence of a very similar concept in the Italian translation of the items (i.e., the concept of checking health/changes in health). The two verbs “to monitor” and “to watch” can be translated in Italian with the same verb, since in Italian there is no distinction between the two verbs. This result was consistent with the finding reported in the German validation study [11], where the inclusion of the covariances between these two items improved the model, as well. Covariances were added also between the residuals of the item 3 and those of the item 37 (“I get enough chances to talk about my health”).

Finally, covariances were added between the residuals of the item 34 (“My health problems do not ruin my life”) and those of the item 39 (“I do not let my health problems control my life”), which both loaded on 4. Constructive attitudes and approaches. It could be hypothesized that in the Italian translation the meaning of the term “problems” can determine the overlapping of the content in the two items.

Reliability, assessed as internal consistency, was acceptable for 1. Positive and active engagement in life, 4. Constructive attitudes and approaches, 3. Skill and technique acquisition, and 6. Health service navigation subscales. It was good for 2. Health directed activities, 8. Emotional distress, and 7. Social integration and support subscales. It should be noted that reliability for 5. Self-monitoring and insight was not acceptable and requires further evaluation.

Evidence about concurrent validity suggested that higher constructive attitudes and approaches were associated moderately with better physical functioning, perceived general health, vitality, social functioning, perceived mental health and lower role limitations due to physical and emotional problems. Higher self-monitoring and insight, health directed activities and social integration and support were associated with higher physical functioning, vitality and perceived mental health. Higher emotional distress was related to lower perceived general mental health, physical and social functioning.

However, concurrent validity was not supported by the absence of correlation between scores on 7. Social integration and support subscale and those on the SF-36 social functioning and by the almost weak correlation between emotional distress and perceived mental health.

### Comparison with other transcultural validations

Overall, the present findings on structural validity appear in line with the validation studies in the other countries and settings [6–12], conducted through highly restrictive tests such as confirmatory factor analyses, which suggested that the questionnaire performs well, the eight scales are distinct, despite it should be noted that the data rarely supported perfect fit and some items present intercorrelated residuals.

Values of reliability of the subscales were consistent with those shown in all the validation studies, conducted in Australia, Germany, Norway, The Netherlands, and France [6–12]. The reliability value did not appear acceptable for 5. Self-monitoring and insight subscale and this result may be considered in line with the original study where it was borderline and also with the studies on other languages where it was not acceptable [6].

Consistent with the study hypotheses and previous evidence [11], to support convergent and divergent validity, moderate associations emerged between 1. Positive and active engagement in life, 4. Constructive attitudes and approaches, and 7. Social integration and support, moderate associations between 3. Skill and technique acquisition and 6. Health service navigation were found, and weak associations between 8. Emotional distress and all the other self-management skills emerged.

Overall, the findings about concurrent validity were in line with the study of Schuler et al. [11] showing that 8. Emotional distress was negatively correlated with all the dimensions of perceived health status assessed by the SF-36 with correlations ranging from − 0.34 to − 0.62. The present results were consistent with that study [11] reporting moderate associations between 1. Positive and active engagement in life, 4. Constructive attitudes and approaches, and (to a lesser extent) 3. Skill and technique acquisition and general health, social functioning, vitality and mental health outcomes. The present findings were in line also with the study by Schuler et al. [11] where 5. Self-monitoring and insight, 6. Health service navigation and 2. Health directed activities were generally only weakly associated with health status outcomes.

In conclusion, the use of the heiQ may be introduced in Italian clinical contexts. For example, it may be useful for practitioners in healthcare services to assess whether a patient with a chronic condition needs to be included in a health education program focused on improving specific self-management skills with the aim to personalize care pathways.

### Limits and future directions

A first issue concerns the heterogeneity of the sample which included patients with different chronic diseases and the relatively limited number of participants. Future research should provide additional evidence about the factor invariance of the scale across different diagnoses and should explore the model fit in other chronic conditions such as chronic pain diseases which are affected by a severe impairment in perceived health status [27, 28]. In addition, despite acceptable fit was reached for the CFI and the TLI indices, as their values were equal to or higher than .90, good fit would require values equal to or higher than .95. Reliability indices were not acceptable for 5. Self-monitoring and insight subscale. Finally, other measures of concurrent validity may be used in further studies such as questionnaires aimed to assess health literacy, health locus of control, optimism, and self-efficacy [29, 30].

## Conclusions

The present study confirmed that the heiQ has strong properties and appears suitable for the assessment of self-management skills in adults with chronic conditions also in Italian healthcare settings.

## Data Availability

The datasets during and/or analysed during the current study available from the corresponding author on reasonable request.
